# The ensemble learning model is not better than the Asian modified CKD-EPI equation for glomerular filtration rate estimation in Chinese CKD patients in the external validation study

**DOI:** 10.1186/s12882-021-02595-5

**Published:** 2021-11-09

**Authors:** Li Zhao, Jing-jing Zhang, Xin Tian, Jian-min Huang, Peng Xie, Xiang-zhou Li

**Affiliations:** 1 Department of Laboratory Medicine, The Third Hospital of Hebei Medical University, Shijiazhuang, 050051 People’s Republic of China; 2 Department of Nuclear Medicine, The Third Hospital of Hebei Medical University, Shijiazhuang, 050051 People’s Republic of China; 3 Department of Nuclear Medicine, The First Affiliated Hospital of Zhengzhou University, NO.1, East Jian-she Road, Zhengzhou, Henan Province 450052 People’s Republic of China

**Keywords:** Chronic kidney disease (CKD), Glomerular filtration rate (GFR), Asian modified CKD-EPI equation, Ensemble learning model

## Abstract

**Objective:**

To assess the clinical practicability of the ensemble learning model established by Liu et al. in estimating glomerular filtration rate (GFR) and validate whether it is a better model than the Asian modified Chronic Kidney Disease Epidemiology Collaboration (CKD-EPI) equation in a cohort of Chinese chronic kidney disease (CKD) patients in an external validation study.

**Methods:**

According to the ensemble learning model and the Asian modified CKD-EPI equation, we calculated estimated GFR_ensemble_ and GFR_CKD-EPI_, separately. Diagnostic performance of the two models was assessed and compared by correlation coefficient, regression equation, Bland–Altman analysis, bias, precision and P_30_ under the premise of ^99m^Tc-diethylenetriaminepentaacetic acid (^99m^Tc-DTPA) dual plasma sample clearance method as reference method for GFR measurement (mGFR).

**Results:**

A total of 158 Chinese CKD patients were included in our external validation study. The GFR_ensemble_ was highly related with mGFR, with the correlation coefficient of 0.94. However, regression equation of GFR_ensemble_ = 0.66*mGFR + 23.05, the regression coefficient was far away from one, and the intercept was wide. Compared with the Asian modified CKD-EPI equation, the diagnostic performance of the ensemble learning model also demonstrated a wider 95% limit of agreement in Bland-Altman analysis (52.6 vs 42.4 ml/min/1.73 m^2^), a poorer bias (8.0 vs 1.0 ml/min/1.73 m^2^, *P* = 0.02), an inferior precision (18.4 vs 12.7 ml/min/1.73 m^2^, *P* < 0.001) and a lower P_30_ (58.9% vs 74.1%, *P* < 0.001).

**Conclusions:**

Our study showed that the ensemble learning model cannot replace the Asian modified CKD-EPI equation for the first choice for GFR estimation in overall Chinese CKD patients.

**Supplementary Information:**

The online version contains supplementary material available at 10.1186/s12882-021-02595-5.

## Introduction

Chronic kidney disease (CKD) is a kind of troublesome disease threatening global human health [1]. According to the latest report, globally in 2017, there were 697.5 million cases of CKD, and almost a fifth of patients with CKD lived in China (132.3 million) [2]. Most patients are asymptomatic at early stage, thus diagnosed at end-stage of the disease, which lead to renal failure and related fatal complications [3]. Therefore, accurate renal function assessment is crucial for early diagnosis, treatment adjustment, and prognostic management of CKD patients. Glomerular filtration rate (GFR) has been considered as the best indicator of renal function assessment and inulin clearance is the gold standard for GFR estimation. However, inulin clearance is inconvenient in practice and with high cost, many alterative algorithms traceable GFR have been established [4]. Among them, the creatinine-based equations, such as modified diet in renal disease (MDRD) and chronic kidney disease epidemiology collaboration (CKD-EPI) equations, have the highest acceptability because of the simplicity and practicability [5–9]. The CKD-EPI equation developed in 2009 was widely used for GFR assessment and outperformed than the others [5, 6]. However, this equation could not adjust for racial variation and may underperform among Chinese CKD patients. Therefore, investigators developed a modified CKD-EPI formula and could correct for Asian race variables [7]. Previous study showed that the Asian modified CKD-EPI equation could achieve a more accurate GFR estimation than the CKD-EPI equation developed in 2009 in Chinese CKD patients [8, 9].

A new ensemble learning model established by Liu *et.al* in 2017, including three variables of sex, age and serum creatinine concentration (Scr), defining the average of an artificial neural network (ANN), support vector machine (SVM), regression equation values as the approximate GFR_ensemble_, provided an alternative [10]. According to the survey, more than 80% of clinical laboratories now provide an approximate GFR when serum creatinine is measured [11]. However, a wrong estimation is worse than none. At present, it is not clear whether the ensemble learning model is a better model than the Asian modified CKD-EPI equation for GFR estimation in Chinese CKD patients. Here we evaluated the comparative performance of the two equations for GFR estimation in Chinese CKD populations, to provide the valuable information for clinical practice.

## Materials and methods

### Ethics statement

The study protocol was approved by Hebei Medical University ethical committee (NO. 2017–027-1), and the written informed consent was obtained from each participant.

### Study subjects

Those subjects following the criteria were enrolled in the study cohort: (1) Chinese patients meeting the diagnostic standard for CKD according to the National Kidney Foundation–Kidney Disease Outcomes Quality Initiative (K/DOQI) clinical practice guidelines [12, 13]; (2) at least 18 years of age. Patients with acute kidney function deterioration, edema, cardiac insufficiency, pleural or abdomen effusion, disabled limb, and treated with cimetidine or trimethoprim or replacement therapy were excluded [5].

### Laboratory measurement

mGFR measurement by the ^99m^Tc-DTPA dual plasma sample clearance method.

The ^99m^Tc-diethylenetriaminepentaacetic acid (^99m^Tc-DTPA) dual plasma sample clearance method was employed as the reference method for GFR estimation (mGFR). ^99m^Tc-DTPA was prepared 30–60 min prior to injection using a current DTPA kit (ShiHong Pharmaceutical Center, Bei Jing, P. R. China). Instant thin-layer chromatography was performed on all DTPA preparations confirming the labeling efficiency> 98%. A dose of 175 MBq ^99m^Tc-DTPA was administered followed by 10 ml sodium chloride 0.9% flush. Residues in dose apparatus and the injection site were assessed using a scintillation probe. If the cumulative residue for an individual patient exceeded 1% of the total dose, then the procedure was considered void and was repeated in full in other day. Heparin anti-coagulated blood samples were taken 2 and 4 h after injection from the opposite forearm. Plasma was separated (3 ml anti-coagulated blood centrifuged for 15 min at a speed of 1500 g), and radioactivity in the plasma (1 ml) was counted in multi-function well counter (CRC-25R multi-function instrument from CAPINTEC.INC, USA). The clearance of ^99m^Tc-DTPA was calculated from a single exponential derived from the blood samples between 2 and 4 h after injection, ^99m^Tc-DTPA plasma clearance (Cl′)was calculated [14]: Cl′ = [D*ln(P_1_/P_2_)]/(t_2_-t_1_)*exp.[(t_1_*ln(P_2_)-t_2_*ln(P_1_))/(t_2_-t_1_)], where D: dosage of drug injected; T_1_: time of first blood sample (about 2 h); P_1_: plasma activity at T_1_; T_2_: time of second blood sample (about 4 h); P_2_: plasma activity at T_2_. Units for D, P_1_, and P_2_ were cpm/ml; units for T_1_, T_2_ was minute. Decay of radioactivity was corrected: Corrected radioactivity = Measured activity*exp. (−ln (2) *interval/6.02). Then, the calculated plasma clearance (Cl′) was corrected by Brochner-Mortensen’s formula [15], GFR = 0.990778Cl’ - 0.001218Cl’^2^ The corrected clearance (GFR) was also standardized for a BSA of 1.73 m^2^(mGFR), according to the Haycock formula [16] of BSA(m^2^) = 0.024265*Wt^0.5378^*Ht^0.3964^, using the patients’ height (cm) and weight (kg) chrematistics.

### The measurement of serum creatinine

The Scr was automatically measured by the enzymatic method on an automatic biochemical analyzer (AU-5821, Beckman company, USA). And the results of Scr were recalibrated with isotope dilution mass spectrometry. The detailed procedure was as in our previous work [17, 18].

### GFR measurement by the ensemble learning model (GFR_ensemble_)

GFR of the ensemble learning model (GFR_ensemble_), is an average value of outputs of ANN(O_ANN_), SVM(O_SVM_) and regression (O_regression_) equation. The three models were constructed using Scr, age, and sex as covariates and GFR as output. Detailed calculation procedure of ANN and SVM models were shown in the additional files 1 and 2 in the Liu *et.al*^,^s article, and the regression model used in the ensemble learning model was shown in Table 1.The ensemble learning model calculation formula was as follows [10]:

$${GFR}_{ensemble}=\frac{0_{ANN}+{0}_{SVM}+{0}_{regression}}{3}$$

**Table 1:** The regression model used in the ensemble learning model [10]

Gender	Scr(mg/dl)	Equation for GFR estimation (Age, years)
Female	≤1.2	92*(Scr/1.2)^-0.534^*(0.994)^age^
	>1.2	79*(Scr/1.2)^-0.516^*(0.994)^age^
Male	≤1.0	98*(Scr/1.0)^-0.450^*(0.996)^age^
	>1.0	105*(Scr/1.0)^-0.640^*(0.993)^age^

Scr represented the serum creatinine concentration

### GFR measurement by the Asian modified CKD-EPI equation (GFR_CKD-EPI_)

The Asian modified CKD-EPI equation was shown in Table 2 [7].

**Table 2:** The Asian modified CKD-EPI equation [7]

Gender	Scr(mg/dl)	Equation for GFR estimation (Age, years)
Female	≤0.7	151*(Scr/0.7)^-0.328^*(0.993)^age^
	>0.7	151*(Scr/0.7)^-1.210^*(0.993)^age^
Male	≤0.9	149*(Scr/0.7)^-0.415^*(0.993)^age^
	>0.9	149*(Scr/0.7)^-1.210^*(0.993)^age^

Scr represented the serum creatinine concentration

### Statistical analysis

Continuous variables conforming to normal distribution were described as mean ± standard deviation (SD); otherwise, by median and interquartile (P_25_–P_75_). Categorical variables were described as frequency and percentage (%).

The relationship between GFR_ensemble_/GFR_CKD-EPI_ and mGFR was assessed with the Spearman correlation analysis and linear regression method. The Bland–Altman method was applied to evaluate the degree of agreement between GFR_ensemble_ / GFR_CKD-EPI_ and mGFR. The comparative performance indicators of GFR estimation for the ensemble learning model and the Asian modified CKD-EPI equation included bias, precision and accuracy. Bias and precision were defined as the median and the interquartile range (IQR) of the difference of GFR_ensemble_/GFR_CKD-EPI_ minus mGFR, respectively. The percentage of GFR within 30% deviation of mGFR (P_30_) was employed as accuracy. And, respective 95% confidence intervals (95%CI) were calculated by means of bootstrap methods (2000 bootstraps) [19]. Wilcoxon signed rank test was performed to compare the bias between the two models, whereas bootstrap method for precision comparison, and McNemar test for comparison of P_30_. All statistical analysis was performed using IBM SPSS statistics 21.0 (IBM Corp., Armonk, NY, USA), MATLAB software (version 2020b, MathWorks) and MedCalc application (version 4.3, Medcalc software, Mariekerke, Belgium). *P* value was two sides and *P* < 0.05 was considered to be statistically significant.

## Results

### Characteristics of the study populations

We collected a total of 192 CKD patients with ^99m^Tc-DTPA dual plasma sample clearance method for GFR estimation, whereas 7 patients lacking of Scr data, age and weight characteristics, 3 patients less than 18 years, 5 patients undergoing dialysis, 3 patients taking drugs effecting serum creatine value, 4 patients with edema and cardiac insufficiency, and 12 patients belonging to outliers after the outliner analysis. Totally, 158 patients were enrolled in our study cohort, 52 cases were chronic glomerulonephritis, 36 cases of diabetic nephropathy, 30 cases of chronic pyelonephritis, hypertensive nephropathy in 21 cases, and other causes or unknown causes in the remaining 19 cases. The basic characteristics of the patients was shown in Table 3.

**Table 3:** Basic characteristics of study populations

Variables	Overall Patients(*n* = 158)
Males, n (%)	73(46.2%)
Age, years, X (SD)	56.5(15.1)
Height(cm), X (SD)	165.3(7.8)
Weight(kg), X (SD)	68.7(13.5)
Serum creatinine (mg/dL), M(P_25_-P_75_)	1.3(0.9–2.3)
mGFR (ml/min/1.73m^2^), M(P_25_-P_75_)	47.7(25.3–80.5)
GFR_ensemble_ (ml/min/1.73m^2^), M(P_25_-P_75_)	56.5(24.5–84.7)
GFR_CKD-EPI_ (ml/min/1.73m^2^), M(P_25_-P_75_)	57.2 (38.1–77.5)

GFR_ensemble_-estimated glomerular filtration rate by the ensemble learning model

GFR_CKD-EPI_ -estimated glomerular filtration rate by the Asian modified CKD-EPI equation

mGFR- glomerular filtration rate determined by ^99m^Tc-DTPA dual plasma sample clearance method

### The validation of the ensemble learning model

**Fig. 1:**
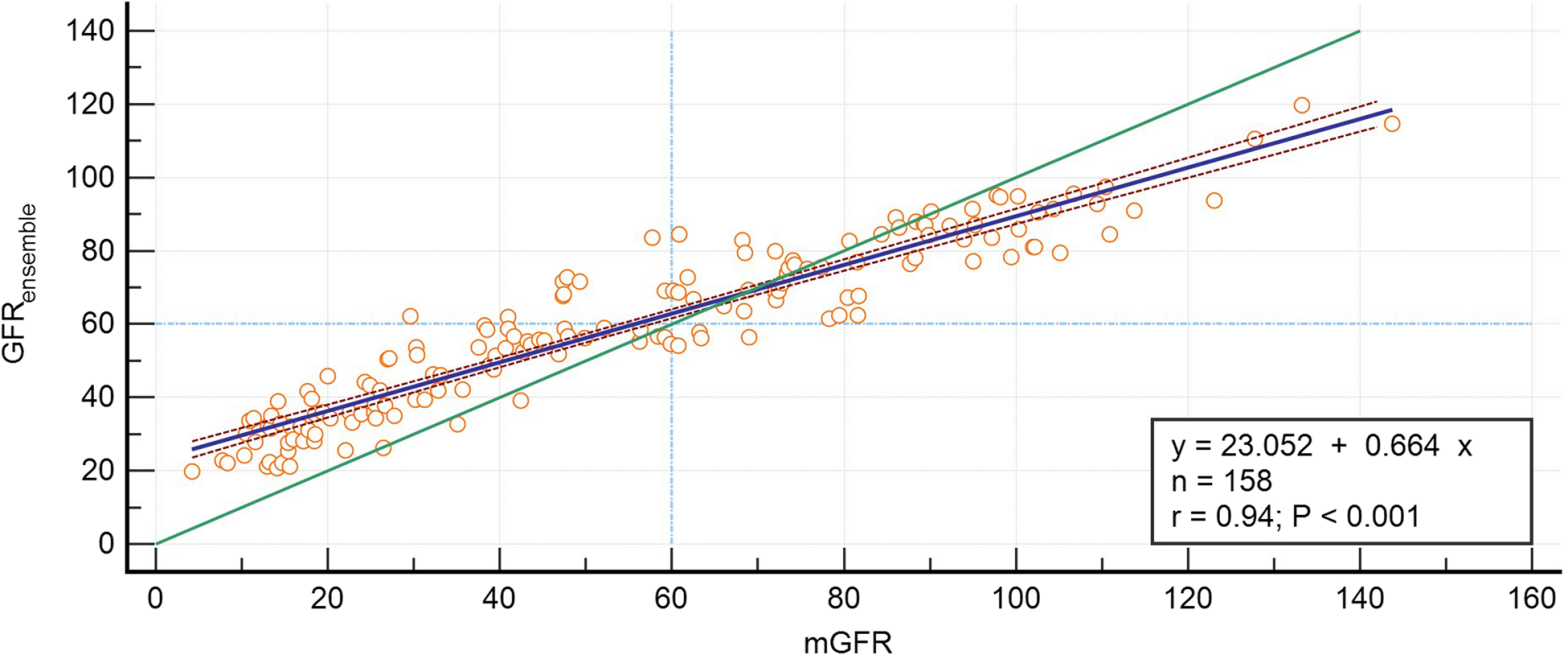
Scatter plots and regression equation of *GFRensemble* and mGFR (ml/min/1.73m^2^). The mGFR was located on the X axis, and the *GFRensemble* was located on Y axis. The solid blue line represented the regression line between *GFRensemble* against mGFR, dashed red lines represent 95% confidence intervals for the regression line. The solid green line represented the identity line of y = x, the two dashed light blue lines were a vertical and horizontal reference line at 60 ml/min/1.73m^2^, respectively

**Fig. 2:**
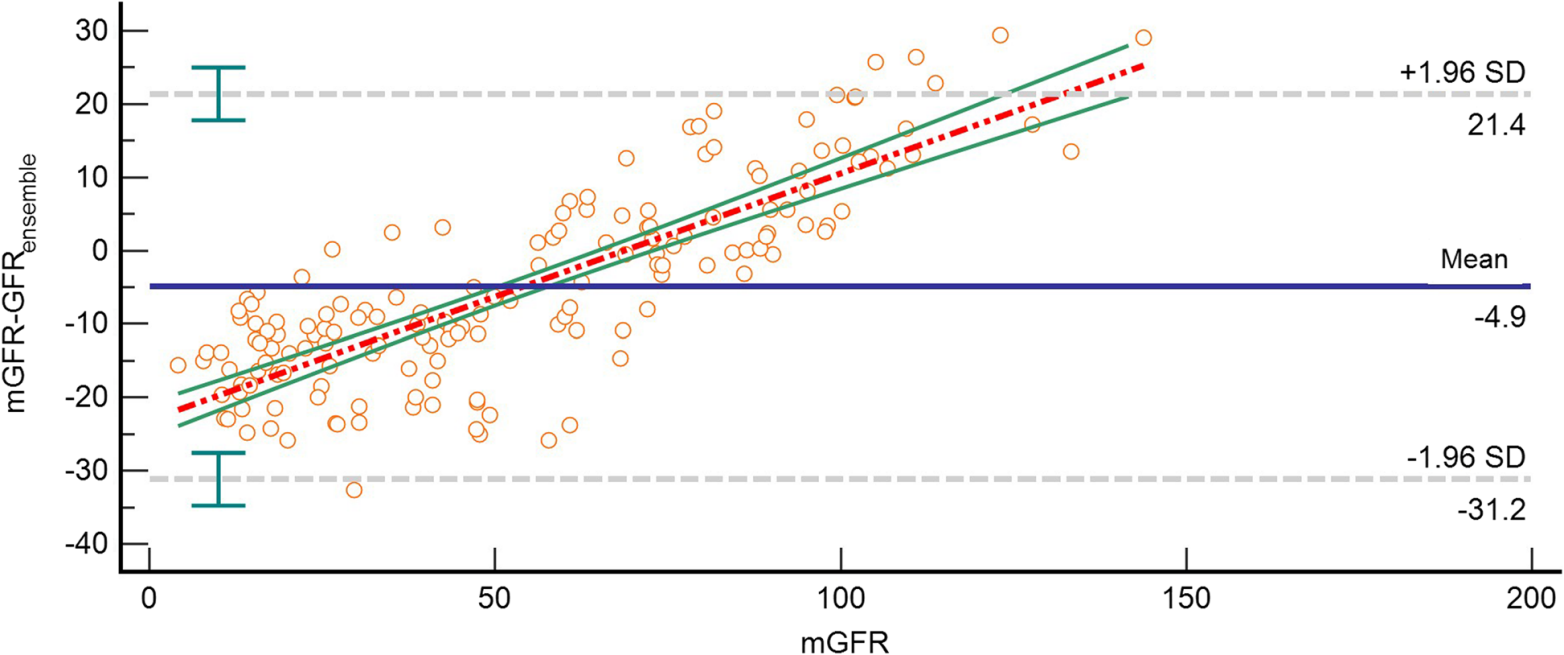
Bland–Altman plot of *GFRensemble* and mGFR (ml/min/1.73 m^2^). The mGFR was located on the X axis, and the value of mGFR minus *GFRensemble* was located on the Y axis. Solid blue line represented the mean of difference between methods, dashed gray lines represented 95% limits of agreement of the mean of difference between methods, dotted red line represented the regression line of the difference between methods against mGFR, solid green lines represented 95% confidence intervals for the regression line

The scatter diagram showed great linear correlation relationship between GFR_ensemble_ and mGFR, with correlation coefficient of r = 0.94 (*P* < 0.001). However, regression equation of GFR_ensemble_ = 0.66*mGFR + 23.05, the slope was away from one and the intercept was too wide. According to the identity line and a vertical and horizontal reference line at 60 ml/min/1.73m^2^, we found that the ensemble learning model might overestimate GFR when < 60 ml/min/1.73m^2^, and underestimate when > 60 ml/min/1.73m^2^ (Fig. 1). The Bland–Altman plot showed the 95% limit of agreement for the ensemble learning model was − 31.2 to 21.4 ml/min/1.73 m^2^ (Fig. 2).

### Performance comparison of the ensemble learning model and the Asian modified CKD-EPI equation for GFR estimation

**Fig. 3:**
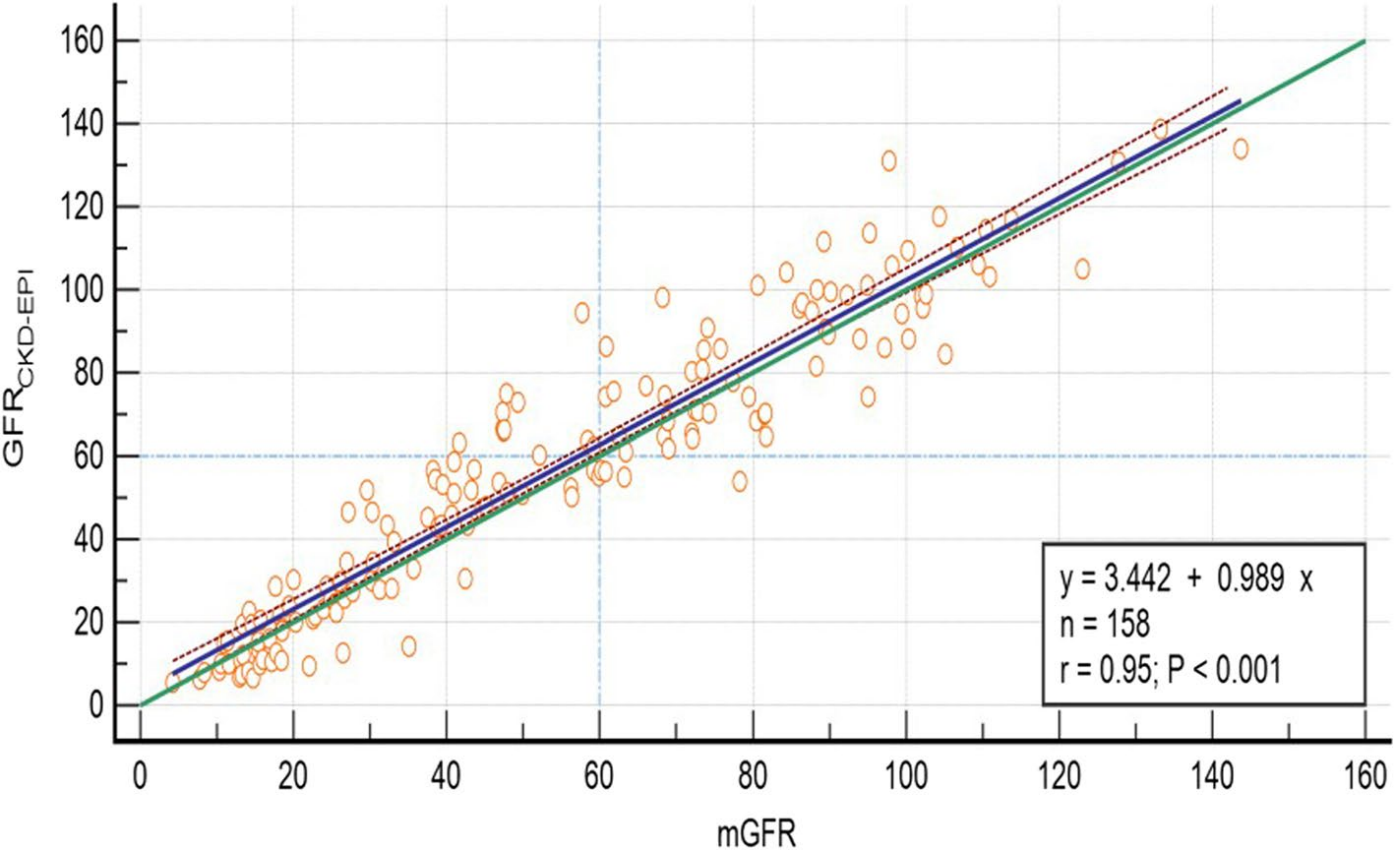
Scatter plots and regression equation of *GFRCKD-EPI* and mGFR (ml/min/1.73m^2^). The mGFR was located on the X axis, and the *GFRCKD-EPI* was located on Y axis. The solid blue line represented the regression line between *GFRCKD-EPI* against mGFR, dashed red lines represented 95% confidence intervals for the regression line. The solid green line represented the identity line of y = x, the two dashed light blue lines were a vertical and horizon reference line at 60 ml/min/1.73m^2^, respectively

**Fig. 4:**
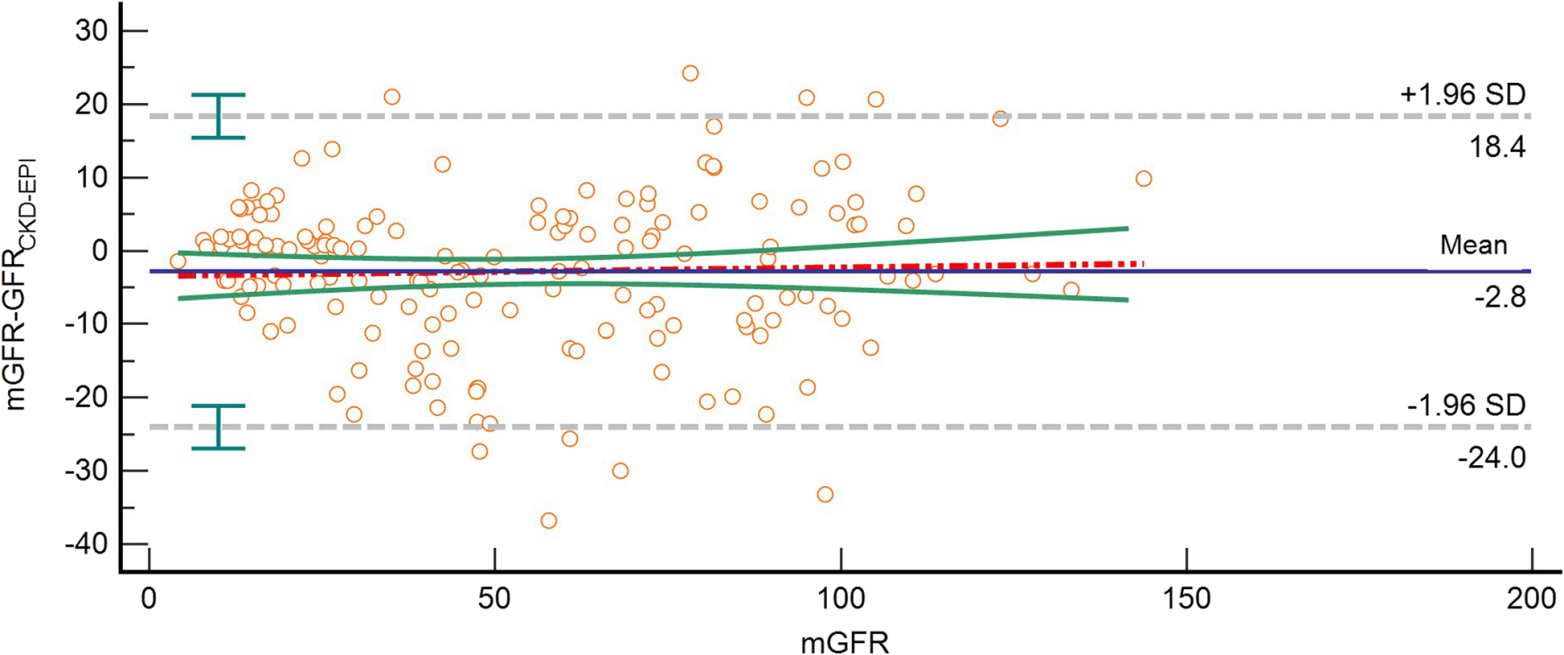
Bland–Altman plot of *GFRCKD-EPI* and mGFR (ml/min/1.73m^2^). The mGFR was located on the X axis, and the value of mGFR minus *GFRCKD-EPI* was located on the Y axis. Solid blue line represented the mean of difference between methods, dashed gray lines represented 95% limits of agreement of the mean of difference between methods, dotted red line represented the regression line of the difference between methods against mGFR, solid green lines represented 95% confidence intervals for the regression line

The correlated coefficient and regression equation between GFR_CKD-EPI_ and mGFR were r = 0.95(*P* < 0.001) and GFR_CKD-EPI_ = 0.99*mGFR + 3.44 (Fig. 3). The Bland–Altman plot showed the 95% limit of agreement for the Asian modified CKD-EPI equation was − 24.0 to 18.4 ml/min/1.73 m^2^ (Fig. 4). Compared with the Asian modified CKD-EPI equation, the slope (0.66 vs 0.99), intercept (23.05 vs 3.44 ml/min/1.73 m^2^) of the regression line, and 95% limit of agreement of the ensemble learning model were all inferior.

The ensemble learning model had a wider bias (8.0[3.4–10.0] vs 1.0[− 0.6–3.8]ml/min/1.73m^2^, *P* = 0.02) and a worse precision (18.4[15.3–21.1] vs 12.7 [9.9–15.0] ml/min/1.73m^2^, *P* < 0.001), and an inferior P_30_ (58.9% [73.7–79.5%] vs 74.1% [67.1–81.0%], *P* < 0.001). The performance of the two models was summarized in Table 4.

**Table 4:** Performance comparison of the Asian modified CKD-EPI and the ensemble learning model

Indicators	Overall (ml/min/1.73m^2^)
**Bias—median difference (95% CI)**	
The Asian modified CKD-EPI equation	1.0^+^ [−0.6–3.8]
The ensemble learning model	8.0^+^ [3.4–10.0], P* = 0.02
**Precision- IQR of the difference (95% CI)**	
The Asian modified CKD-EPI equation	12.7 [9.9–15.0]
The ensemble learning model	18.4[15.3–21.1], P* < 0.001
**Accuracy—P**_**30**_**(95% CI)**	
The Asian modified CKD-EPI equation	74.1% [67.1–81.0%]
The ensemble learning model	58.9% [73.7–79.5%], P* < 0.001

P*-denoted the comparison was statistically significant between GFR_ensemble_ and GFR_CKD-EPI_

+ − denoted the median of bias of the two equations

## Discussion

Considering the clinical limitation of inulin as the gold standard for GFR assessment in the CKD patients, new algorithms were constantly developed to approximately estimate GFR. The ensemble learning model established by Liu *et.al* in 2017 generated an alternative [10]. There was no literature about whether the ensemble learning model was accurate and suitable for external populations. In our current study, we assessed the performance of the ensemble learning model by these indicators of correlated coefficient, regression equation, Bland-Altman analysis, bias, precision, and P_30_ under the premise of ^99m^Tc-DTPA dual plasma sample clearance method as reference method for GFR estimation, and compared with the creatine-based Asian modified CKD-EPI equation.

Our study showed the ensemble learning model was not a better model for GFR estimation in overall Chinese CKD patients. In our external validation cohort, in spite of the high correlation and relatively great 95% limit of agreement with mGFR, both the slope (0.66) and the intercept (23.052) of the ensemble learning model was unsatisfactory. Our findings were discrepant from Liu et al^’^s research, we demonstrated a wider bias (8.0 vs 2.3 ml/min/1.73m^2^), an inferior precision (18.4 vs 14.0 ml/min/1.73m^2^), and a worse P_30_ (58.9% vs 75.1%) than the primary study results of Liu et al. Furthermore, in our current study, compared with Asian modified CKD-EPI equation, the ensemble learning model also had a more positive bias, significantly overestimating the real GFR of CKD patients, and a lower P_30_, evidently decreasing the estimation accuracy.

The reason why the performance of the ensemble learning model is worse in our external validation populations may focus on the following three points. Firstly, Liu et al. employed the ^99m^Tc-DTPA renal dynamic imaging method as the reference to establish the three ANN, SVM and regression equation. Numerous researches have demonstrated renal dynamic imaging method is not suitable as the reference standard because of its unsatisfactory performance [20]. In our validation cohort, we used the ^99m^Tc-DTPA dual plasma clearance method as the reference standard. The average Scr was 1.7 ± 1.8 mg/dl with a mean GFR of 70.0 ± 29.6 ml/min/1.73m^2^ in the development dataset and Scr was 2.7 ± 2.5 mg/dl for a mean GFR of 53.4 ± 26.5 ml/min/1.73m^2^ in Liu et al*’s* validation dataset showed in Table 2. However, in the current study, the median Scr was 1.3 (0.9–2.3) mg/dL for a median GFR of 47.7 (25.2–80.5) ml/min/1.73m^2^. An unrealistic higher GFR at high Scr in the Liu et al ‘s article led to more errors were introduced in the establishment of the three equations, which may account for this poor performance of the ensemble learning model in our external validation study in significant sense. The bias, precision, and P_30_ of the Asian modified CKD-EPI equation in our validation article was similar with results in the primary 4-levels CKD-EPI algorithm external validation [7]. So, our validated results maybe more accurate. Secondly, the regression equation used in the ensemble learning model was established by Liu et al.*,* which may compromise the assessment results. In the phrase of data analysis, we indeed found regression algorithm adopted by the ensemble learning model was more biased than the Asian modified CKD-EPI equation. Existing researches have shown the Asian modified CKD-EPI equation is the most accurate liner regression equation in predicting GFR. Furthermore, we found that the bias of ANN and SVM equations of the ensemble learning model were almost the same, all greater than the Asian modified CKD-EPI. Therefore, the ensemble learning model was not only algorithmically complex, but also poorly accurate. Thirdly, due to different populations recruited in separate study, the validated results may be different.

Actually, the global performance of the Asian modified CKD-EPI equation remained poor (P_30_ = 74.1%) in our validated cohort. The GFR estimation result is not ideal, the clinicians needed evaluate the accuracy of the GFR equations when applying them in a different population in the clinical practice firstly.

The present study was small sample size and only CKD patients were included, therefore, the effectiveness of the ensemble learning model still need more external validation in other centers in other population by more sample size.

## Conclusion

The ensemble learning model was not a better model for GFR estimation in overall CKD patients in our external validation cohort, not only having complex calculation but also poor accuracy. However, the global P_30_ of the Asian modified CKD-EPI equation remains 74.1%, the clinician should assess the GFR of CKD patients based on the patient’s actual situation in combination with the equation in the clinical practice.

## Supplementary Information

**Additional file 1.** (XLS 55 kb)

**Additional file 2.** (XLS 179 kb)

## Data Availability

The datasets used and/or analyzed during the current study are available from the corresponding author on reasonable request.

## Abbreviations

CKD: Chronic Kidney Disease
GFR: Glomerular Filtration Rate
MDRD: Modified Diet in Renal Disease
CKD-EPI: Chronic Kidney Disease-Epidemiology Collaboration
ANN: Artificial Neural Network
SVM: Support Vector Machine
^99m^Tc-DTPA: ^99m^Tc-diethylenetriaminepentaacetic Acid
Scr: Serum creatinine
IQR: Interquartile Range
